# 

**Published:** 1917-11

**Authors:** 


### ANNOUNCEMENTS

OHIO STATE DENTAL SOCIETY. The Fifty-first Annual Meeting of the Ohio State Dental Society will be held in Cleveland, December 4, 5, and 6, in the Hollenden Hotel.

The program promises one of the greatest and best dental meetings ever held. Among the speakers already secured are: E. C. Rosenow, M.D., "Mouth Infections Relating to Systemic Diseases;" Dr. Truman W. Brophy, "Fractures of the Jaws;" Dr. John P. Buckley, "The Treatment of Pulpless Teeth; and Technic of Cleaning and Filling of the Canals;" Dr. N. S. Hoff, "Impression Taking;" Dr. R. H. Riethmueller, "Dangers of and Sequellae of Conductive Anesthesia;" Dr. G. F. Fette, "Ionization;" Dr. W. H. O. McGehee, "Dental Caries;" Dr. Carl Lucas, "Periodontaclasia."

A new feature under the supervision of Dr. Weston A. Price will be a "seminar" covering the entire field of non-vital teeth. "Evidence, not opinion, to be used," and to be comprised in a series of twelve ten-minute discussions, further exemplified by progressive clinics.

Other clinics will cover the field of the management of vital teeth for supporting artificial substitutes.

There will be no general discussions, the entire time being devoted to papers, lectures and clinics.

The display of dental supplies will be unusually complete, and Tuesday forenoon and Thursday afternoon have been set aside as special exhibitor's sessions.

Come for the first day's program and bring note books and pencil. F. R. Chapman Secretary, 305 Schultz Bldg., Columbus, Ohio.

IOWA STATE BOARD OF EXAMINERS. The next meeting of the Iowa State Board for the examination of applicants will be held at Iowa City, Iowa, commencing December 3, at 9:00 a. m.

For further information address the secretary, Dr. J. A. West, 417 Utica Bldg., Des Moines, Iowa.

